# Proteome investigation of an organellar fraction of *Toxoplasma gondii*: a preliminary study

**DOI:** 10.1186/1753-6561-8-S4-P74

**Published:** 2014-10-01

**Authors:** Davi da S Ferreira, Iane T Menezes Resende, Jorge A Lopez

**Affiliations:** 1 Universidade Tiradentes, Aracaju, Sergipe, 49032-490, Brazil; 2 Programa de Pós-Graduação em Biotecnologia Industrial, Universidade Tiradentes/Instituto de Tecnologia e Pesquisa, Aracaju, Sergipe, 49032-490, Brazil

## Background

*Toxoplasma gondii *is a ubiquitous Apicomplexan parasite responsible for systemic diseases in both humans and animals. Toxoplasmosis is a major public health problem, infecting one-third of the world's human population and leading to abortion in domestic animals [1]. The search for new chemotherapeutic targets is imperative, due to its increasing resistance to the drugs currently available for combating this parasite [2]. Recent high-throughput

Proteomic approaches have provided a wealth of protein expression data on Apicomplexan parasites (*e.g., T. gondii, Plasmodium falciparum*), while a number of smaller-scale studies have examined specific drug-related hypotheses. Proteomic methods can be applied to study sub-cellular localization, cell function, organelle composition, changes in protein expression patterns in response to drug exposure, drug-protein binding, and validation of data from genomic annotation and transcript expression studies [3]. Organellar structures have therefore become potential targets for the parasite life cycle to control the levels of nutrients or salts that surround them [4]. The aim of this study was to perform a proteomic analysis of an organellar fraction of this Apicomplexan protozoan based on the structural and metabolic aspects.

## Methods

An acidocalcisome (AC) organellar of *T. Gondii *RH strain tachyzoites was obtained by an iodixanol density gradient [4]. The AC fraction was digested with trypsin and further analyzed by liquid chromatography-mass spectrometry (LC-MS) due to its potential for metabolite screening in proteomic studies. LC-MS analysis was conducted using a nanoHPLC coupled to an ESI-TOF-MS instrument. Trypsin-digested samples were loaded into a pre-column (Reversed phase C_18_) and desalted with 0.1% formic acid. Peptide separation was performed in a capillary column (PepMap, C_18 _5 μm 300 Å, 75 μm I.D., 15 cm). Elution was carried out using a linear gradient (5-40% of solvent B) for 200 min (solvent A = 0.1% formic acid; and solvent B = 90% acetonitrile/0.08% formic acid). The eluate was introduced into the nanospray source set with 5.3 kV of ionization energy and a desolvation temperature of 150°C. Scans were acquired every second throughout the 400 to 2000 a.m.u. range. The precursor (doubly- and triply-charged) ions with intensity above 10 counts were dynamically selected and subjected only once to collision-induced dissociation for 4s, using the default automatic rolling collision energy. The MS/MS scans were acquired throughout the 50 to 1800 a.m.u. range. 108 proteins were confidently identified by data-mining *T. gondii*-specific databases.

## Results and conclusions

Although some proteins indicate contamination from other cell compartments, around 50% of the proteins were exclusively identified from the AC fraction (*e.g*., kinases, an ATP/ATP-carrier protein, a GTP-binding-like protein, lysophospholipase) and other proteins with no annotated function or homology. Although preliminary, these organellar proteins may represent data to be analyzed and converted into metabolic information in future studies for experimental investigation, and potentially suitable targets for the development of therapeutic strategies, in addition to the role of proteomics approaches in the biomedical area [3,5].
